# Effects of Supplementary Feeding on the Breeding Ecology of the Buff-Throated Partridge in a Tibetan Sacred Site, China

**DOI:** 10.1371/journal.pone.0146568

**Published:** 2016-01-19

**Authors:** Nan Yang, Timothy C. Moermond, Huw Lloyd, Yu Xu, Liang Dou, Kai Zhang, Bisong Yue, Jianghong Ran

**Affiliations:** 1 Key Laboratory of Bio-Resources and Eco-Environment of Ministry of Education, College of Life Sciences, Sichuan University, Chengdu 610064, China; 2 Division of Biology and Conservation Ecology, School of Science and the Environment, Manchester Metropolitan University, Chester Street, Manchester, United Kingdom; Cornell University, UNITED STATES

## Abstract

Our goal was to document effects of year-round supplemental feeding on breeding ecology of the Buff-throated Partridge, *Tetraophasis szechenyii*, within a Tibetan sacred site. We evaluated effects of supplemental feeding used as religious/cultural practices which could potentially aid conservation of endangered phasianids. We compared fed breeding groups to neighboring nonfed groups. Fed groups initiated first clutches significantly earlier than nonfed groups. Earlier laying groups within fed and nonfed groups showed significantly lower hatching rates than later groups; however, fed groups showed significantly higher hatching rates than nonfed groups laying in the same period. Earlier laying increased opportunities to renest. All six fed groups with clutch failures renested compared to only one of five nonfed groups with clutch failures. Fed female breeders showed significantly greater investment in their young with larger clutches and larger eggs, which likely increased survivability of early hatchlings. We observed no predation on birds at feeding sites and recorded only four cases of predation on incubating females, which showed no detectable difference between fed and nonfed groups. Ground-nesting birds typically face high risks of predation. Ten of the 48 groups nested in trees, which occurs in few phasianid species. Tree nests showed significantly higher hatching rates compared to ground nests; however, we found no significant difference in tree nesting between fed and nonfed groups. This partridge is one of four gallinaceous species with cooperative breeding. Breeding groups with helpers had significantly greater reproductive success than single pairs, and fed female breeders with helpers laid bigger eggs than single pairs. Comparing annual reproductive output per group, fed groups not only produced significantly more independent young (≥150 days post-hatching), their young hatched significantly earlier, which likely have greater reproductive value over later hatched young of nonfed groups. Supplemental feeding year-round is likely what enabled the successes of the fed partridges.

## Introduction

This study began out of interest and concern for the Buff-throated Partridge (*Tetraophasis szechenyii* Madarász), which is an unusual, little known, and endangered “pheasant-partridge” endemic to high altitude tree-line regions of western China [1,2]. This species is of special concern as it has undergone a dramatic regional population decline due to the degradation and loss of tree-line habitats and illegal hunting in many areas throughout western China [2–4]. This study was first initiated at a high altitude alpine site, the Pamuling sacred site, where monks of the Pamuling monastery have promoted protection of local wildlife and have provided supplemental food daily the year around for Buff-throated Partridges and two other pheasant species (Blood Pheasant, *Ithaginis cruentus*, and White Eared Pheasant, *Crossoptilon crossoptilon*). As a result, the partridges that live within this sacred site and receive daily supplemental food have become habituated to the presence of humans and, therefore, were much easier to observe than wild partridges. At this Pamuling sacred site, it is this special religious practice of year-round supplemental feeding which made this study possible and which may serve as a potentially valuable practice for the conservation of rare pheasants like the Buff-throated Partridge.

The provision of food for gallinaceous species is a traditional practice employed by indigenous communities within cultural protected areas across many Himalayan regions, particularly within the Tibetan sacred sites [5]. These sacred sites are ecologically unique areas, which occur in areas of high species richness and endemism within biodiversity hotspots [6–10]. The hunting of wildlife and selective logging are prohibited within Tibetan sacred sites and forest preservation is strongly promoted [9,11]. Consequently, these Tibetan sacred sites provide an additional level of protection for populations of several high-elevation montane pheasant species, which are threatened elsewhere by habitat loss and hunting [2,4,12].

Supplemental feeding regimes are known to have multiple effects on their target populations and that responses tend to vary widely for different kinds of birds [13–17]. The positive effects of supplemental feeding have included advances in clutch initiation dates [13,14,17–33], increased chances for renesting or 2^nd^ clutches [16,22,23,30], increases in clutch size and egg size [5,25,28,30,34], improved body condition and survival [16,18,30,35–38], increases in reproductive success and productivity [21,30,32,39,40–42], increased fitness value of early hatched young [22,24,26,30,43], and reduced risk of predation [13,35,36,44]. Other avian studies, however, have reported a number of adverse effects of supplemental feeding regimes [14,30] including reduced brood size and lower hatching success [22,26,32,45], side-effects like male biased sex ratio [46], reduced organ and gut size [47], increased risk of disease transmission [48], and increased risk of predation [49].

The principal goal of this study is to assess the effects of year-long supplemental feeding on the breeding ecology and reproductive success of the Buff-throated Partridges living in the Pamuling sacred site. In this particular case, the breeding ecology of these partridges included two special characteristics nearly unique among gallinaceous species: nesting in trees [50] and cooperative breeding [51].

These partridges typically roost together in trees [52–54], and, although most groups nest on the ground, some groups in the Pamuling Mountain area have constructed their nests in trees. Roosting in trees occurs in a number of gallinaceous species, but nesting in trees is very rare among pheasants, reported only in the two *Tetraophasis* and five *Tragopan* species [50,55,56]. Both the group “communal” tree roosting [54,57] and the tree nesting are behaviors expected to reduce predation risk, which is expected to be important for this species given that predation is the greatest cause of mortality in such ground-dwelling phasianid species [15].

The Buff-throated Partridge is a regular cooperative breeder [51], which is extremely rare amongst the Galliformes. Only three other species of the Galliformes engage in cooperative breeding [58–60]. These partridges’ cooperative breeding groups are typically small, resident territorial groups composed of one breeding pair and one to three adult “helpers”, usually males [51,54]. This partridge population also includes “groups” of two composed of a single breeding pair, which is characteristic of regular cooperatively breeding species [61]. Cooperative breeding among bird species often increases foraging efficiency and survival, but no evidence of this had been found for these partridge cooperative breeders before this study. [51].

## Methods

### Ethics statement

The study was carried out on data we collected on Pamuling Mountain, and we received the necessary permission for working at the Pamuling scared site by the Pamuling Monastery. All the field work followed the regulations of the Protection of Wildlife Law of the People’s Republic of China. All our observational and field studies and lab work were approved by the Wildlife Protection Office of the Sichuan Provincial Forestry Department and by the Ethics Committee of Sichuan University, China.

### Study site

**Fig 1:**
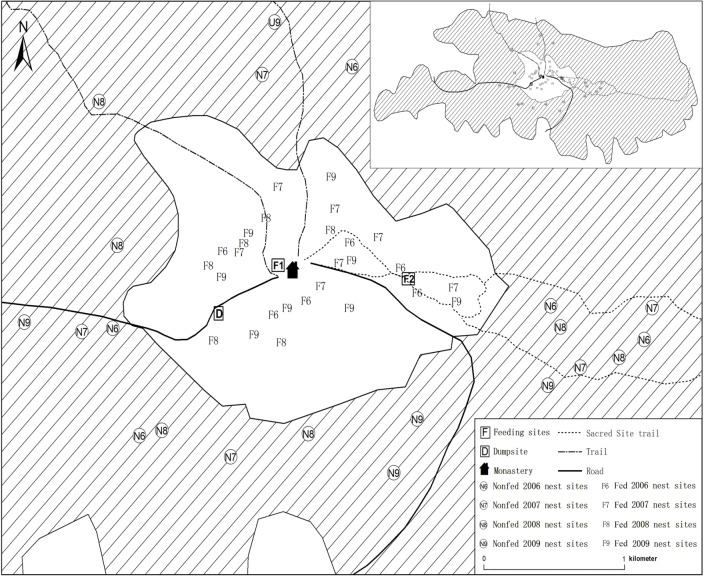
Map of the study area showing the monastery location, feeding site locations, dumpsite, and locations of all fed and nonfed group nest sites for each year. The light gray central area is the Pamuling sacred site. The larger gray hatched area is the top of Pamuling Mountain from 3350m up (see the entire area from 3350m up in the inset).

Our study was conducted over a four-year period from March 2006 through December 2009 on Pamuling Mountain (30°06′N 101°11′E) in the Ganzi Tibetan Autonomous Prefecture, Sichuan Province, China. Our study site encompassed the top of the Pamuling Mountain from the highest peak at 4400 m down to an elevation of 3350 m which defined the lower limit of the study area. The 3350 m elevation limit serves as an outer boundary which circumscribes the entire 50.6 km^2^ study area as shown in Fig 1. The light gray area in the center delimits the boundary of the 339 ha Pamuling Tibetan Monastery sacred site within which the supplemental feeding sites are located (Fig 1). The entire area shown in Fig 1 is covered with a mosaic of Himalayan tree-line habitats. The dominant vegetation of the sacred site and the surrounding area above 3350m was *Quercus aquifolioides* oak forest on the southern and western slopes of the area, with flaky fir, *Abies squamata*, forest and violet-purple rhododendron, *Rhododendron nitidulum*, scrub (approximately 50 cm in height) dominating north-facing slopes. Alpine meadows, primarily composed of Sichuan kobresia (*Kobresia setchwanensis)* made up a smaller proportion of the tree-line mosaic [51,62]. The semi-humid climate was typical of the Qinghai-Tibetan plateau tree-line with spring occurring from April to June, summer only in July, autumn from August to September, and a long winter from October to March [62].

### Study species

Our study species, the Buff-throated Partridge and its congener, the Chestnut-throated Partridge (*Tetraophasis obscurus*), are high altitude pheasants (also called pheasant-grouse, pheasant-partridge, and monal-partridge) which are closely related to the monal pheasants (*Lophophorus* spp.) and tragopans (*Tragopan* spp.) [63–65]. Despite their close phylogenetic relations to these other pheasants, these *Tetraophasis* pheasants likely were called ‘partridges’ due to their cryptic plumage and lack of sexual dimorphism. The plumage patterns of these two *Tetraophasis* species are characteristic of pheasant species that are monogamous with multi-year pair bonds [55]. Their monogamous habits with longer term pair bonds, cryptic plumage, territorial behavior, communal roosting, occasional tree nesting, and cooperative breeding all taken together suggest a special adaptive model for a species faced with a short breeding season, a long cold high altitude winter, and a likely prevalent risk of predation [50–52,54,62].

The wild foraging (not supplemented) partridge breeding groups living in the surrounding area beyond the central sacred site fed mainly on plant roots, stems, flowers, and occasional insects [2,66]. Cooperative breeding territorial groups typically spend all their time together and use the same roost site year-round [52–54]. The breeding female of each group typically lays three to four eggs per clutch (range 1–5), which are incubated exclusively by the dominant breeding female [50]. Within the Pamuling Mountain study area, we selected two different sets of breeding groups: (1) fed groups, which received daily supplemental food (typically rice and corn) within the vicinity of the Monastery, and (2) nonfed (i.e., non-supplemented) groups that foraged exclusively on natural resources. All the fed groups, including their territories [62], were found only within the area of the Pamuling sacred site (i.e., the central core area shown in light gray in Fig 1). The nonfed groups all were found in the surrounding areas outside of the central Pamuling sacred site. Locations of all the nest sites for fed and nonfed groups for the four years of the study are shown on the map in Fig 1. The locations of the nonfed groups’ nest sites are indicated by small rings with an ‘N’ and year (i.e., ‘06’, ‘07’, ‘08’, or ‘09’), and locations of the fed groups’ nest sites are indicated by an ‘F’ and year as noted above. Supplemental feeding occurred at three known fixed sites around the Monastery. The two main supplemental feeding sites were two ‘ritual offering’ sites (F1 and F2 in Fig 1), where each morning monks from the Pamuling Tibetan Monastery provided rice and corn. The food at these two sites was augmented sporadically throughout the day by offerings contributed by local people from the neighboring community when they worshipped at the sacred site. The third supplemental feeding site was a dumpsite (D in Fig 1), where the monks from the monastery threw “household” garbage which included food waste from the kitchen and canteen. The two partridge groups that fed at this site over the four years of the study [62] were also treated as “fed” groups; however, we only found and monitored nests of these two territorial groups in 2008 and 2009. Both the supplemental fed and the nonfed breeding groups occupied areas with a similar composition of tree-line habitats [62].

Birds were observed every day from mid-March to late-July, corresponding to the breeding season, and from mid-August to late-October (and occasionally later), which corresponded to the first half of the non-breeding season. Each spring, the field team searched for and found as many breeding group nest sites as possible when the breeding females were laying their eggs. Although a total of 68 fed groups had been recorded over the four years within the core sacred site shown in Fig 1, finding active nests of fed groups was not easy. Yet, finding nests of nonfed groups was much more difficult, since nonfed groups were spread out over a much larger area and were far more wary of humans. We found and observed active nest sites for 27 supplemental fed groups (six in 2006, seven in 2007, six in 2008, and eight in 2009) and 21 nonfed groups (five in 2006, five in 2007, six in 2008, and five in 2009). Members of the same breeding family group were confirmed based on observations that all members foraged together and shared the same roosting tree [52,54].

Once a nest was found, it was examined two days later to determine the clutch initiation date, since eggs were usually laid around mid-day [67]. Following completion of the clutch, nests were visited every two days to determine whether breeding attempts were on-going or whether the nests had failed. Since incubation periods typically varied from 20 to 25 days, at 17 or 18 days after the clutch initiation date, we began checking the nests every day to record the hatching date. From these observations, we were able to determine clutch completion date, clutch size, incubation period, and hatching date. Bird remains and/or large numbers of feathers at the nest-site during the breeding period were taken to indicate that the breeding female had been preyed on [5].

In order to set up comparable subject cases for calculating reproductive success [68], we chose the start of incubation as the starting point for the comparative study of the fed and nonfed groups. Therefore early nests that were terminated before egg-laying was completed due to the death of the breeding female or simply deserted were not considered in the analyses of reproductive output; however, breeding females killed by predators during incubation were included.

As part of the analyses of reproductive success, for each clutch, we recorded the number of eggs (clutch size) and egg size. We measured the maximum length and breadth of each egg to the nearest mm (or occasionally nearest 0.5 mm) with a band tape and weighed each egg to the nearest gram (or occasionally nearest 0.5 g) using a spring balance. When incubation was completed, we recorded the hatching date and the number of chicks hatched as well as the number of unhatched eggs. For purposes of assessing different aspects of reproductive “success”, “hatchlings” were considered as equivalent to “fledglings”, since the partridge “hatchlings” are precocial. Chicks that survived to 150 days post-hatching were considered to be “independent young” with similar morphometric measurements to the adults [67]. We defined “hatching rate” of a clutch as the number of hatchlings over the total number of eggs in the clutch including unhatched or lost eggs. We defined “clutch failure” as meaning the loss (or failure to hatch) of *all* the eggs in a clutch and “brood failure” as the loss (death or disappearance) of *all* the chicks from a given clutch.

We considered total “annual reproductive success” as measured by three different outputs: the total number of eggs, hatchlings, and independent young produced per breeding group with the number of independent young considered as the most important output [69]. For any group that renested in the same season, the output of both the first and second clutches were added together to represent the “total annual reproductive success” of that group.

### Selection of study groups and possibility of pseudoreplication

Longitudinal observational studies, such as ours, raise the possibility of pseudoreplication from year to year [70], which assumes “the same groups occur in the same treatments year after year”. Although 35 fed partridges were banded as part of several overlapping field studies, only eight banded birds occurred in fed groups of this study. Those eight were distributed among six fed nesting groups. None of the eight banded birds were present in a breeding group in more than two consecutive years, although identification of repeat individuals allowed one group to be identified in a third year. Therefore, we could confirm that six fed groups were directly related to seven later groups, which involved a total of 13 groups, 48% of the 27 fed groups. For the nonfed groups, no birds were banded; however, two groups were identified from individual characteristics of group members to have been the same group in a subsequent year, which involved only four groups, 19% of the 21 nonfed groups.

Examination of the seven cases among the fed groups where we knew that a group in one year was present in the following year, the seven cases showed a total of 13 important changes: 3 changed group size, 5 new adult male helpers appeared of which 4 came from other groups (only one new male helper was recruited from the progeny of that group), 2 breeding males were replaced the following year by an outside male that previously had been a helper in that group, and 3 changed their nest type (2 tree to ground nest; 1 ground to tree nest). Similarly, for nonfed groups, the two cases identified also showed important changes: one with a change in group size, the other with a replacement of a male helper with a male from another group. The prevalence of these year-to-year changes among the breeding groups of those nine cases diminishes the concern about pseudoreplication.

Also, in addition to the observed year-to-year changes in group sizes and composition, we never found a breeding group using the same nest site the following year; therefore, locations of the nest sites discovered each year all differ from the previous year's nest sites (see the locations of all the nesting sites in Fig 1). This means that 1) each group changed its nest site from year to year, and 2) it was often not possible to find the nest of the same group each year. The result is that there were many cases where group nesting sites from a given year, say 2006, did not show any nearby nesting sites for the next year (i.e., 2007 in this example), in which case, it is unlikely that those breeding groups were represented in the following year.

The search in early spring each year to locate nesting sites was spread across different areas. Note in the map in Fig 1 how the locations of nesting sites for fed groups are well distributed over the central sacred site (light gray area), and the nonfed groups are widely distributed in the neighboring areas surrounding the central sacred site (gray hatched area). The relatively wide scattering of the nesting sites each year acted to increase the likelihood of including a broader, non-selective sampling of the groups within the study area. Given these points, we conclude that the possible problem of pseudoreplication is small and very likely outweighed by the changes in group size and composition and in nest type and location as well as by other factors and chance occurrences related to weather, predation, etc.

### Data analysis

As a precautionary measure to avoid possible complications of pseudoreplication, we used Fisher’s exact test to determine the probability of most of the differences between groups we examined. Fisher’s exact test is a nonparametric one-tailed statistical test which determines the probability that the differences as defined with respect to a specific hypothesis among two cases compared in a 2X2 table could be the same due to chance.

The additional parametric statistical tests used for a few special analyses were described as follows. A General Linear Model (GLM) univariate test was used to compare the differences in laying dates of first clutches of fed and nonfed groups with year as a random factor. We used analysis of covariance (ANCOVA) to assess the effects of food on egg size (egg length, breadth, and weight) with clutch size as a covariate and on clutch size with clutch initiation date as a covariate. In all these statistical tests, we used p ≤ .05 as indicating statistical significance. The parametric statistical tests were conducted using SPSS for windows release 17.0 (SPSS Inc. 2001, Chicago).

## Results

### Early laying by the supplemental fed breeding groups

Early laying is the single most common effect from supplemental feeding [13,14,16,22]. Fed groups laid first clutches significantly earlier than nonfed groups (*p* < 0.001; Table 1) with their mean laying date (April 9) being 19 days earlier than the mean laying date for nonfed groups (April 28; Table 1). Although the laying dates varied from year to year, the GLM-univariate test with food as a fixed factor and year as a random factor, showed an effect for food but not for year.

**Table 1:** Laying dates of first clutches and number of replacement clutches of fed and nonfed groups.

H1a^1^, Fed groups bred earlier than nonfed groups		Fed groups (27)	Nonfed groups (21)	GLM-univariate test
	Mean ± SE	14.3 ± 1.7	33.2 ± 1.6	*F* = 92.5; *p* < 0.001
	(1 = Mar 27)	(14 = Apr 9)	(33 = Apr 28)	
	Range	Mar 27-Apr 19	Apr 13-May 12	
H1b^1^, Fed groups bred earlier than nonfed groups		Fed groups (27)	Nonfed groups (21)	Fisher’s exact test
	1^st^ clutch laid > Apr 15^2^	24 (89%)	1 (5%)	*p* < 0.001
	1^st^ clutch laid < Apr 15	3	20	
H2, More fed groups with 1^st^ clutch failures renested compared to nonfed groups		Fed groups with failed clutches (6^3^)	Nonfed groups with failed clutches (5)	Fisher’s exact test
	# Groups that renested	6 (100%)	1 (20%)	*p* = 0.015
	# Groups did not renest	0	4	

^1^ This hypothesis was tested by two different statistical tests: ‘a’ indicates a parametric test; ‘b’ indicates a nonparametric test (Fisher’s exact test).

^2^ We visually chose the break point date near the joint median point that maximized the contrast between fed and nonfed laying dates.

^3^ One fed group whose entire brood failed the day after hatching was included as a “failed clutch” in this case.

One of the common benefits of earlier laying is an increase in the opportunity to renest, particularly in the case of failed clutches [16,30]; therefore, we expected fed groups with clutch failures to be more likely to renest compared to nonfed groups (H2). We recorded six clutch failures among fed groups, which included one group whose brood failed the day after hatching. All six of these fed groups renested. We recorded five clutch failures among nonfed groups, but only one renested and that one failed. The difference was significant (*p* = 0.015; Table 1).

Given the long winters on Pamuling Mountain, we expected that early laying carried greater risks of clutch failure due to extreme weather and low food availability. We, therefore, expected the earliest laying fed groups to have lower hatching success than later laying fed groups (H3) and, also, expected the earliest laying nonfed groups to have lower hatching success than later laying nonfed groups (H4). However, if the supplemental food gave fed groups greater ability to deal with the early spring cold, we expected that the later laying fed groups would show higher hatching rates than the earliest laying nonfed groups during the same time period (H5). All three of these early/late breeding comparisons showed significant differences (Table 2).

**Table 2:** Hatching rates of early and late layers within and between fed and nonfed groups.

H3, Late laying fed groups had greater hatching rates than early laying fed groups		10 Late Fed groups (Apr 12–19)^1^	17 Early Fed groups (Mar 27 -Apr 11)^1^	Fisher’s exact test
	# Hatchlings	26 (68%)	32 (48%)	*p* = 0.032
	# Eggs lost	12	35	
H4, Late laying nonfed groups had greater hatching rates than early laying nonfed groups		11 late Nonfed groups (Apr 27 -May 12)^1^	10 Early Nonfed groups (Apr 13–26)^1^	Fisher’s exact test
	# Hatchlings	26 (72%)	15 (44%)	*p* = 0.016
	# Eggs lost	10	19	
H5, For groups laying at same period, fed groups had greater hatching rates than nonfed groups		10 Late Fed groups (Apr 12–19)	10 Early Nonfed groups (Apr 13–26)	Fisher’s exact test
	# Hatchlings	26 (68%)	15 (44%)	*p* = 0.033
	# Eggs lost	12	19	

^1^ We divided early and late nonfed groups (10:11) as equally as possible. We then chose the number of late fed groups (10) that laid 1^st^ clutches within the time period of early nonfed groups (10). That left the early fed groups which all laid before any nonfed groups as the largest set of groups (17).

### Increased clutch and egg sizes in fed groups

Supplemental feeding occurring in spring before the time of egg laying (or year-round feeding, as in this study) has shown increased clutch and egg sizes in some cases [16,30,32,42]. Therefore, we assumed that first clutches of fed groups would be larger than for nonfed groups (H6a,b). Although the first clutches of fed groups were on average a half egg larger, an ANCOVA (H6a) failed to show a statistically significant difference (*p* = 0.15); however, a simple Fisher’s exact test (H6b) showed that 78% of the fed groups had first clutches of 4 or 5 eggs compared to only 38% for nonfed groups, which was a significant difference (*p* = 0.006; Table 3).

**Table 3:** First clutch sizes of fed groups compared to nonfed groups.

H6a^1^, Fed 1^st^ clutches larger than nonfed 1^st^ clutches		Fed groups (27)	Nonfed groups (21)	ANCOVA
	Mean clutch ± SE	3.89 ± 0.1	3.33 ± 0.1	*F* = 2.2; *p* = 0.15 NS
	Range	3–5	2–4	
H6b^1^, Fed 1^st^ clutches larger than nonfed 1^st^ clutches		Fed groups (27)	Nonfed groups (21)	Fisher’s exact test
	Clutch of 4–5 eggs	21 (78%)	8 (38%)	*p* = 0.006
	Clutch of 2–3 eggs	6	13	

^1^ This hypothesis was tested by two different statistical tests: ‘a’ indicates a parametric test; ‘b’ indicates a nonparametric test (Fisher’s exact test).

We also expected that egg sizes (lengths and breadths) and weights would be larger in fed groups than in nonfed groups (H7a,b; H8a,b; & H9a,b).The differences for all three aspects of egg size were highly significant as shown both by the ANCOVA’s and Fisher’s exact tests (Table 4).

**Table 4:** Egg sizes in first clutches of fed groups compared to nonfed groups.

H7a^1^, Fed egg lengths greater than nonfed eggs		Fed groups (26)^2^: 100 eggs	Nonfed groups (21): 70 eggs	ANCOVA
	Mean egg length ± SE	54.6mm ± 0.2	51.9mm ± 0.3	*F* = 45.6; *p* < 0.001
	Range	49–58mm	42–55mm	
H8a^1^, Fed egg breadths greater than nonfed eggs		Fed groups (26)^2^: 100 eggs	Nonfed groups (21): 70 eggs	ANCOVA
	Mean egg breadth ± SE	37.3mm ± 0.2	35.0mm ± 0.2	*F* = 55.0; *p* < 0.001
	Range	34–42mm	33–38mm	
H9a^1^, Fed egg weights greater than nonfed eggs		Fed groups (25)^3^: 95 eggs	Nonfed groups (19)^3^: 64 eggs	ANCOVA
	Mean egg weight ± SE	37.4g ± 0.4	33.5g ± 0.2	*F* = 41.7; *p* < 0.001
	Range	33–41g	30–36.5g	
H7b^1^, Fed egg lengths greater than nonfed eggs		Fed groups (26)^2^	Nonfed groups (21)	Fisher’s exact test
	# Eggs > 54mm^4^	82 (82%)	13 (19%)	*p* < 0.001
	# Eggs ≤ 54mm	18	57	
H8b^1^, Fed egg breadths greater than nonfed eggs		Fed groups (26)^2^	Nonfed groups (21)	Fisher’s exact test
	# Eggs > 37mm^4^	73 (73%)	10 (17%)	*p* < 0.001
	# Eggs ≤ 37mm	27	60	
H9b^1^, Fed egg weights greater than nonfed eggs		Fed groups (25)^3^	Nonfed groups (19)^3^	Fisher’s exact test
	# Eggs > 36g^4^	80 (84%)	1 (1.6%)	*p* < 0.001
	# Eggs ≤ 36g	15	63	
H10, Fed groups mean egg weights per clutch greater than for nonfed groups		Fed groups (25)^3^	Nonfed groups (19)^3^	Fisher’s exact test
	Mean clutch wt > 35.5g^4^	22 (88%)	0 (0%)	*p* < 0.001
	Mean clutch wt ≤ 35.5g	3	19	
H11, Fed 1^st^ clutches total weight > nonfed 1^st^ clutches (egg weight X clutch size)		Fed groups (25)^3^	Nonfed groups (19)^3^	Fisher’s exact test
	Clutch total wt > 136g^4^	19 (76%)	2 (11%)	*p* < 0.001
	Clutch total wt ≤ 136g	6	17	

^1^ This hypothesis was tested by two different statistical tests: ‘a’ indicates a parametric test; ‘b’ indicates a nonparametric test (Fisher’s exact test).

^2^ Egg lengths and breadths were measured for the clutches of only 26 of the 27 fed groups.

^3^ Egg weights were recorded for the clutches of only 25 of the 27 Fed groups and for only 19 of the 21 Nonfed groups.

^4^ Egg lengths, breadths, and weights of fed and nonfed groups were compared by visually choosing break points near the joint median point that maximized the contrast between fed and nonfed values.

We also wanted to compare differences in egg sizes at the scale of individual first clutches as shown by group mean egg weights (H10). The difference was highly significant: 88% of the fed groups had greater mean egg weights than nonfed groups (Table 4). In addition, the total investment by weight (clutch size X mean egg weight per group) was significantly greater for fed breeding females compared to nonfed breeding females (Table 4).

**Fig 2:**
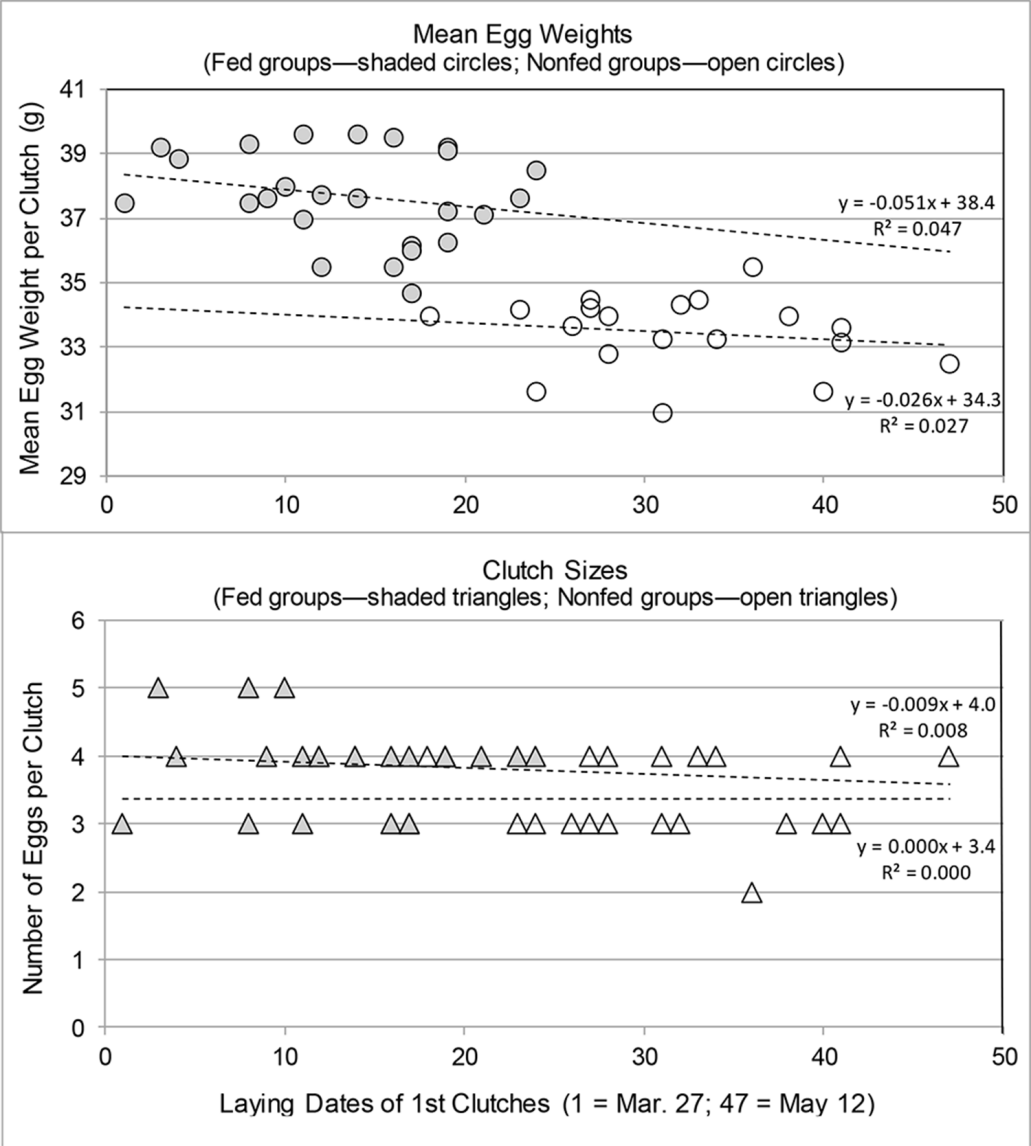
Comparison of first clutch sizes and mean egg weights of fed and nonfed groups by laying dates.

Given that some studies have reported seasonal variation in clutch sizes and egg sizes relative to early or late laying dates [26], we looked at the possible seasonal relationship of clutch sizes and mean egg weights with respect to laying times of first clutches by graphing the variation in these two key variables over the full time span of first clutch laying dates for both fed and nonfed groups (Fig 2). Although first clutch sizes for fed groups were shown to be significantly greater than for nonfed groups (Table 3), variation in first clutch sizes showed virtually no detectable differences related to laying dates for either fed or nonfed groups as shown by the linear regression lines for fed and nonfed clutches. Mean egg weights showed a slight decline of about a one gram difference between the first to last dates for both fed groups and nonfed groups (Fig 2). Note that the fed group mean egg weights were consistently greater than the nonfed group mean egg weights with almost no overlap.

Among fed groups, the renesting clutches were significantly smaller (1–3 eggs, mean renest clutch = 2.2) than first clutches (*p* < 0.001, Table 5). There were only six renesting clutches with a total of 13 eggs, but only eight eggs from four of the clutches had been measured. The egg sizes from the four renesting clutches of fed groups, as measured by egg weight and egg breadth, were also significantly smaller than first clutches (*p* < 0.001, Table 5); however, the egg lengths of the renesting clutches were marginally different from eggs of first clutches (*p* = 0.053; Table 5, H15). Note that the egg weights of the fed renesting clutches fell in the size range of the nonfed first clutch egg weights.

**Table 5:** Clutch and egg sizes of fed first clutches compared to fed renesting clutches.

H12, Fed 1^st^ clutches larger than fed renest clutches		Fed 1^st^ clutches (27)	Fed renest clutches (6)	Fisher’s exact test
	Clutch of 4–5 eggs	21 (78%)	0 (0%)	*p <* 0.001
	Clutch of 1–3 eggs	6	6	
H13, Fed 1^st^ clutch egg weights > renest egg weights		Fed 1^st^ clutches (25)	Fed renest clutches (4)	Fisher’s exact test
	# Eggs > 36g^1^	80 (84%)	0 (0%)	*p <* 0.001
	# Eggs ≤ 36g	15	8	
H14, Fed 1^st^ clutch egg breadths > renest egg breadths		Fed 1^st^ clutches (26)	Fed renest clutches (4)	Fisher’s exact test
	# Eggs > 37mm^1^	73 (73%)	0 (0%)	*p <* 0.001
	# Eggs ≤ 37mm	27	8	
H15, Fed 1^st^ clutch egg lengths > renest egg lengths		Fed 1^st^ clutches (26)	Fed renest clutches (4)	Fisher’s exact test
	# Eggs > 54mm^1^	82 (82%)	4 (50%)	*p* = 0.053
	# Eggs ≤ 54mm	18	4	

^1^ Egg weights, breadths, and lengths for first and renesting clutches were compared using the same break points that were used to compare eggs of fed and nonfed groups (Table 4).

### Predation at nests on incubating females and on eggs of first clutches

Although we were unable to calculate adult survival for fed or nonfed groups, observations of breeding females during incubation, when these females are considered quite vulnerable [16, 37], provided a well-defined time period (three to four weeks) to allow valid comparisons of mortality of female breeders of fed and nonfed groups. Given the possibility of higher predation risk due to higher densities of fed groups, we expected that fed groups may experience higher predation than nonfed groups (H16). We recorded four breeding females killed during incubation on ground nests by unknown predators: three from fed groups and one from a nonfed group. The difference was not significant with a high *p*-value (*p* = 0.910).

Given that the Buff-throated Partridges at this site were observed to occasionally use tree nests [50], we assumed that tree nests offered greater security against clutch predation (H17). The numbers were too low to determine the hatching success of tree nests versus ground nests within fed groups or nonfed groups; therefore, we combined fed and nonfed groups in order to determine the relative nesting success of groups with tree nests compared to groups with ground nests. All ten tree nests (100%) produced at least one hatchling compared to only 61% of groups with ground nests. The difference was statistically significant (*p* = 0.014; Table 6). Since this test confirmed that tree nests showed a higher hatching success, we assumed that tree nests would be expected to produce more hatchlings per egg than ground nests. The greater numbers involved allowed us to test this hypothesis within fed groups (H18) and nonfed groups (H19). In both cases, tree nests showed significantly higher hatching rates than ground nests (Table 6). Last, we tested whether the use of tree nests was more frequent among nonfed groups than fed groups. That result was not significant (Table 6).

**Table 6:** Hatching success at tree nests versus ground nests for fed and nonfed groups.

H17, Tree nests > success than ground nests (all groups combined)		Tree nests	Ground nests	Fisher’s exact test
1^st^ Clutches	≥ 1 Hatchling	10 (100%)	23 (61%)	*p* = 0.014
	No hatchlings	0	15	
H18, Tree nests had greater hatching rates than ground nests (Fed groups)		Fed tree nests (4 nests)	Fed ground nests (23 nests)	Fisher’s exact test
1^st^ Clutches	# Hatchlings	13 (87%)	49 (54%)	*p* = 0.016
	# Eggs lost	2	41	
H19, Tree nests had greater hatching rates than ground nests (Nonfed groups)		Nonfed tree nests (4 nests)	Nonfed ground nests (23 nests)	Fisher’s exact test
1^st^ Clutches	# Hatchlings	19 (83%)	22 (47%)	*p* = 0.004
	# Eggs lost	4	25	
H20, Nonfed groups nested in trees more often than fed groups		Nonfed groups (21)	Fed groups (27)	Fisher’s exact test
1^st^ Clutches	# Tree nests	6 (29%)	4 (15%)	*p* = 0.210 NS
	# Ground nests	15	23	

### Annual reproductive success as total output per group for fed and nonfed groups

As measures of annual reproductive success, we expected that fed groups produced more eggs (H21), more hatchlings (H22), and more independent young (H23) per group compared to nonfed groups (annual outputs from first clutches and renesting clutches were combined). The ANCOVA analyses showed that fed groups produced significantly more eggs per group compared to nonfed groups (*p* < 0.001; Table 7) but did not confirm that the number of hatchlings per fed group was significantly more than for nonfed groups (*p* = 0.086; Table 7). For the most meaningful measure, the ANCOVA showed that the fed groups produced significantly more independent young per group compared to nonfed groups (*p* = 0.022; Table 7),

**Table 7:** Annual reproductive success as total output per group for fed and nonfed groups.

H21, Fed groups produced more eggs than nonfed groups		Fed groups (27)	Nonfed groups (21)	ANCOVA
	Eggs, Mean ± SE	4.37 ± 0.23	3.38 ± 0.13	*F* = 12.3
	Range	1–5	1–4	
	(Total # / # groups)	(118 / 27)	(71 / 21)	*p* < 0.001
H22, Fed groups produced more hatchlings than nonfed groups		Fed groups (27)	Nonfed groups (21)	ANCOVA
	Hatchlings, Mean ± SE	2.59 ± 0.31	2.0 ± 0.28	*F* = 3.09
	Range	0–5	0–4	
	(Total # / # of groups)	(70 / 27)	(42 / 21)	*p* = 0.086 NS
H23, Fed groups produced more 150 day young than nonfed groups		Fed groups (27)	Nonfed groups (21)	ANCOVA
	Independent young, Mean ± SE	1.11± 0.22	0.81 ± 0.21	*F* = 5.59
	Range	0–4	0–3	
	(Total # / # of groups)	(30/ 27)	(17 / 21)	*p* = 0.022
H24, Fed independent young hatched earlier than nonfed young		Fed groups (27)	Nonfed groups (21)	Fisher’s exact test
	# young hatched before Apr 17^1^	24 (80%)	0 (0%)	*p* < 0.001
	# young hatched after Apr 17	6	17	

^1^ We visually chose the break point date near the joint median point that maximized the contrast between fed and nonfed laying dates.

Several studies have shown that young produced by early breeders are more likely to survive than young produced later in the breeding season [18,22,24]. Given the significant advancement in laying dates shown by the fed groups, we hypothesized that fed groups would produce more young hatched earlier than the young of nonfed groups (H24). We found that 80% of fed young were hatched earlier than any of the young of nonfed groups; the difference was highly significant (*p* < 0.001; Table 7).

### Cooperative groups compared to single pair groups for fed and nonfed groups

Since the nonfed groups appeared to have fewer single pair groups than fed groups, we first tested whether nonfed groups had significantly more cooperative groups (i.e., groups with helpers) than fed groups (H25). The difference was not significant (*p* = 0.381; Table 8). Second, we tested whether the presence of helpers in a breeding group (i.e., a cooperative group) affected the group’s reproductive success compared to single pair groups. We hypothesized (H26) that cooperative groups were more likely to produce at least one independent young than single pair groups. Due to the small sample sizes, we were unable to show statistically significant differences between the groups with helpers compared to single pairs within nonfed groups (*p* = 0.094) or fed groups (*p* = 0.124). Therefore, we then combined all the groups to compare all cooperative groups against all single pair groups. With this larger sample, we were able to show that cooperative groups (i.e., groups with helpers) were significantly more likely to produce independent young than single pair groups (*p* = 0.025; Table 8).

**Table 8:** Cooperative groups compared to single pair groups of fed and nonfed groups.

H25, Nonfed groups are more likely to have helpers than fed groups		Nonfed groups (21)	Fed groups (27)	Fisher’s exact test
	# Groups with helpers	15 (71%)	17 (63%)	*p* = 0.381 NS
	# Single pair groups	6	10	
H26, Cooperative groups (groups with helpers) showed greater breeding success than single pairs (fed & nonfed groups combined)		Coop groups (32)	Single pairs (16)	Fisher’s exact test
	≥ 1 Independent young	21 (66%)	5 (31%)	*p* = 0.025
	No independent young	11	11	

Given that finding, we hypothesized (H27, H28) that breeding females in cooperative groups, with the benefit of one or more adult helpers, would produce larger eggs than breeding females in single pair groups (Table 9). Fed cooperative groups did produce significantly greater mean egg weights in 1^st^ clutches compared to fed single pair groups (*p* = 0.034; Table 9), but differences were not detected in unfed groups (*p* = 0.184; Table 9). We then hypothesized (H29) that the total investment in 1^st^ clutches (clutch size X mean egg weights) of fed cooperative groups would be greater than for fed single pair groups. The total investment of the fed cooperative groups was significantly greater than for fed single pairs.(*p* = 0.006; Table 9). The same comparison applied to the nonfed cooperative groups was not significant (test not shown).

**Table 9:** Egg weights of cooperative groups vs. single pairs of fed and nonfed groups.

H27, Nonfed coop group egg weights greater than nonfed single pair eggs		Nonfed coop groups (14)	Nonfed single pairs (5)	Fisher’s exact test
	Mean egg weight ≥ 34.0g^1^	8 (57%)	1 (20%)	*p* = 0.184 NS
	Mean egg weight < 34.0g	6	4	
H28 Fed coop group mean egg weights greater than fed single pairs		Fed coop groups (16)	Fed single pairs (9)	Fisher’s exact test
	Mean egg weight ≥ 38.0g^1^	9 (56%)	1 (11%)	*p* = 0.034
	Mean egg weight < 38.0g	7	8	
H29 Fed coop groups showed greater total investment in 1^st^ clutches than fed single pairs		Fed coop groups (16)	Fed single pairs (9)	Fisher’s exact test
	Clutch size X egg wt. > 153g^1^	9 (56%)	0 (0%)	*p* = 0.006
	Clutch size X egg wt. < 152g	7	9	

^1^ Egg weights between coop and single pair nonfed groups and fed groups and total clutch weights between coop and single pair fed groups were compared by visually choosing break points near the joint median point that maximized the contrast between coop and single pair values.

## Discussion

Supplemental feeding is about increasing food availability at a local level and is often targeted for particular species, like the Buff-throated Partridge. Our goal was to assess the effects of year-round supplemental feeding on the breeding ecology and reproductive success of the Buff-throated Partridges fed at the Tibetan sacred site on Pamuling Mountain.

The single most common effect of supplemental feeding is earlier breeding, an advancement of laying dates, which effectively increases the length of the breeding season [13,14,16,18,20]. The fed partridges we studied showed a significant shift to early breeding (Table 1) with a mean advancement of 19 days over nonfed groups with *all* the fed groups laying earlier than 86% of the nonfed groups. Compare this with a four-year study of Tibetan Eared Pheasants (*Crossoptilon harmani*) which received supplemental food (highland barley) from late autumn until the start of incubation in late spring at a Buddhist nunnery near Llasa, Tibet [5]. Compared to nonfed pheasants living in the same area, the fed pheasants showed an average advancement of laying dates of only two days, which was not significant [5]. Early laying “buys more time” for second clutches, such as renesting after clutch failure [16,22,23,30]. For the fed partridges, the six females with failed clutches all renested compared to one out of five for nonfed groups (Table 1).

Early laying can lead to an early finish of the breeding season, which would give the parents more time to molt and recover fat reserves before winter [18,21,22,71]. We observed that out of the 27 fed groups, the 18 groups (67%) that bred early and did not renest had significantly more time than nonfed groups, thus giving the fed female breeders the time and resources for a complete molt before winter. In contrast, a late breeding female may have to shorten her molting period, which may leave her with a cheap, poorly insulated ‘coat’, which will generate increased thermoregulatory costs and, therefore, greater risk of mortality as this bird moves into winter [18,71].

Perhaps, the most compelling advantage of early laying is that *fledglings produced by early breeders survive better than fledglings produced later* [24,72]. The increasing mortality rate of young hatched later in the season suggests strong selection for early nesting [44]. Young hatched early in the season are likely to be larger and more experienced and, therefore, often dominant over later hatched young, and will likely have an advantage competing for resources and for positions in winter groups [18,22]. Offspring from first nests would be the oldest members of their cohort and could establish a level of dominance that promotes their ascendancy to breeder status [30,73]. The high reproductive value of early hatched young selects for early laying [18,20,22].

Young hatching earlier have a much higher chance of surviving and demonstrate greater reproductive value [22,24,30]; therefore, a more useful statistic is the relative number of independent young hatched earlier from fed versus nonfed groups. The earlier breeding fed groups not only produced significantly more young, but the most important difference was that 80% of fed young hatched earlier than any nonfed young (Table 8).

What are the limits of earlier advancement of laying? If early laying buys time, what does it cost? On Pamuling Mountain, even with supplemental food, attempting to lay in early April or into March is moving into the end of winter, when it is still quite cold and late snowstorms still happen. We examined the reproductive costs of pushing the limits of how early the partridge bred by comparing success among four different sets of groups: the earliest fed groups (Mar 27-Apr 11); the later laying fed groups (Apr 12–19); the earliest laying nonfed groups (Apr 13–26); and the later laying nonfed groups (Apr 27-May 12). We focused on hatching rates because that stage was most likely to be affected by the conditions during incubation. Because the earliest fed groups suffered more losses and lower hatching rates than the later fed groups and were not clearly any more successful than the earliest nonfed groups, was their higher effort and risk worth it? The benefits of an early timing of breeding can be quantified only when considering also the post-fledging period [74]. We do know that the four fed groups with clutch failures all renested with three producing independent young and that the 12 early independent young hatched in the earliest breeding fed groups may well have had greater reproductive value than the later hatched young of all the later laying groups [44].

Lack of available food is most likely the greatest constraint against early breeding [14,21,22,30,32]. Due to the length and cold of the high altitude winter, the availability of natural foods is low in winter, and high protein foods are likely to be at their lowest at the end of winter and beginning of spring just when early partridge breeders would face their greatest needs.

For precocial species like the partridge, the highest energy and nutritional demands are during egg-laying when their daily expenditure for the production of clutches is greater than for altricial species [16,25]. The high energy demands of laying are followed next by incubation when the breeding females must spend nearly all of their time at the nest sitting on the clutch to protect the eggs from cold and predators. While they are incubating, they have little time to forage, relying instead on their fat reserves [16,37]. To be able to provide that high level of energy into the first two stages of breeding, female pheasant species, such as grouse, ptarmigans, and these partridges, need to be in good condition with sufficient fat reserves *before* they begin to lay their first clutch in early spring [16,37,75,76].

Along with stimulating early breeding in birds, supplemental feeding before and during the breeding season may lead to production of larger clutches and larger eggs [22,30,77]. Both represent greater investments by the female breeder to increase the number and survivability of the young. The fed female partridge breeders significantly increased clutch size more often from three to four (Table 3) and significantly increased the size of their eggs (Table 4). The greater investment in clutch size and, particularly, in egg size showed that the resources available were abundant enough to support those investments. In particular, the relatively consistent amount of the increase in egg size may have been to improve the size and survivability of their chicks which would be hatching under less than favorable environmental conditions of early spring at that high altitude [28,44].

The first clutch sizes of fed and nonfed birds did not decline over the laying period of the first clutches (Mar 27-May 12)(Fig 2). The mean egg weight per group did show a slight decline from early to late laying dates for both fed and nonfed groups, but the R^2^ values were very low (Fig 2). In contrast, the fed partridges’ six renesting clutches were all significantly smaller with smaller egg weights (Table 5). The reduced sizes of these replacement clutches do appear to fit into the general pattern of seasonal decline in clutch size [24–26,28,77]. Larger egg size can produce a larger hatchling, which may give a better chance to survive [28,44]. Smaller egg sizes, in contrast, allow the chick to hatch earlier and may reduce the cost to females [26].

Supplemental feeding also carries some risk associated with a higher density of birds near feeding sites, which is likely to increase competition and may attract predators and increase the risk of predation [13,14,30,49]. The few cases of predation on adult partridges we recorded were at nests, not at feeding sites, and we found no significant difference between fed and nonfed groups.

With predation being a major risk for ground-dwelling gamebirds [15], the partridges in our study engaged in several behaviors to reduce risks from predators. One was communal roosting in trees, in which all members of a group roost together in dense conifer trees, which gives them protection from predators and thermoregulatory benefits through cold nights [52–54,57]. Another was nesting in trees. While roosting in trees occurs in many gallinaceous species, nesting in trees has been reported only in the Buff-throated Partridge and in the five species of tragopans (*Tragopan*) [50,55,56]. In our study, most of the nests were on the ground with just over one-fifth found in trees. Although we found no difference in the use of tree nesting by fed and nonfed groups, we were able to show the first evidence that hatching success of groups that nested in trees was significantly greater than for those groups that nested on the ground (all groups combined, Table 6), and the groups nesting in trees showed significantly greater hatching rates compared to groups nesting on the ground both within nonfed groups and within fed groups (Table 6).

Supplemental feeding increases survival of fed birds; the increase in food availability may free more time for vigilance and reduce exposure to predation [13,18,33,35,36] as well as more time for resting and preening [5,18,36]. Winter food supplementation increases rates of winter survival of game birds [38]. Winter feeding for songbirds (often from fall into early spring) increases productivity in the subsequent breeding season [36,42,78]. For populations of two *Parus* species in Sweden, food supplementation in autumn and winter improved winter survival, which led to a doubling of the fed breeding populations the following spring [35].

Young adult partridges remaining with their parents within the safety of their known territory can gain greater security and experience [78]. At the same time, these young adults benefit the breeding pair by assisting in territorial defense and vigilance for predators and joining in communal roosting [54]; this is the basis of the cooperative breeding shown by Buff-throated Partridges [51]. The mutual fitness benefits of breeding cooperatively are likely to stem from group living per se rather than from alloparental effects [79–81], which is why solitary male partridges often join as a helper to a territorial breeding pair [51], and, in addition to the benefits of group living, unrelated male helpers also increase their chances of becoming the dominant breeding male [82]. Such promotion occurred in two of the seven year-to-year cases of fed groups with marked individuals (described in Methods above). Ours is the first study of these cooperative breeding partridges to show that cooperative breeding groups (i.e., breeding groups with adult helpers) experience greater reproductive success than single pairs. Groups with helpers were significantly more likely to produce independent young compared to single pair groups (Table 8).

Among the partridge cooperative breeding groups, helpers have been observed to contribute to vigilance and territorial defense [51,54]. Such assistance by partridge helpers may give the breeding female more time to feed and accumulate greater resources to lay earlier and to invest more in their clutches [78]. We were able to show that among the supplemental fed groups, breeding groups with helpers produced significantly greater mean egg weights compared to single pair groups (Table 9); however, nonfed groups with helpers did not show a significant difference compared to single pair groups (Table 9). We suggest that the combined benefits of supplemental feeding and assistance of adult helpers work together to further reduce the workload of a fed female breeder and to increase her time and resources compared to fed single pairs or to nonfed female breeders with helpers.

A related study of cooperative breeding in Arabian babblers (*Turdoides squamiceps*) showed that helpers with access to supplemental food were able to spend longer periods on sentinel duty without a loss in body mass [83]. That example shows further how the benefits of supplemental feeding can serve to enhance the benefits provided by helpers in a cooperative breeding group. We suggest that for partridge fed groups, supplemental feeding may reduce feeding time for all adult members of cooperative groups, which may further increase vigilance of helpers, thereby further reducing the risk of predation [54]. For a fed female breeder with helpers, the combined benefits may allow her to invest even more in her offspring without significantly increasing her reproductive costs, as shown by the significantly larger eggs produced by fed females in cooperative groups compared to fed females in single pair groups [Table 9]. We suggest that a more detailed study of vigilance and foraging behaviors of fed cooperative breeding partridge groups would be valuable in many ways.

## Conclusion

It is year-round supplemental feeding that provides a continuous daily support that increases the probability of survival to all the birds in a partridge breeding group. The year-round availability of supplemental food buffers adverse periods, giving a more stable temporal distribution of food as compared with natural conditions [19], thereby favoring long-term success. Florida Scrub-Jays have consistently bred early in suburban areas where human-provided food is ubiquitous year-round, suggesting that resource predictability may be a perceptual cue for the appropriate timing of breeding [33]. The strong annual response of the Pamuling fed partridge groups with exceptionally early breeding, larger clutches, and larger eggs may be due in part a response to the predictability of the long-term year-round supplemental feeding at the Pamuling sacred site.

The fact that the fed partridge groups showed such a strong set of significant responses to the supplemental feeding provides substantial evidence that the particular example of supplemental feeding practiced at the Pamuling Tibetan sacred site appears to be a successful conservation approach. A number of our results, while statistically significant, were based on small sample sizes; therefore, we recommend that the supplemented population should be monitored to assure that the beneficial effects of such a treatment continue [30].

Tibetan sacred sites offer a unique opportunity to continue to explore the many benefits and costs of supplemental feeding as a conservation tool for threatened bird species management. We, therefore, encourage further comparative studies examining differences in resource availability, density, behavior, and survivorship between supplemental fed and nonfed populations of the Buff-throated Partridge and similar pheasant species across a number of sacred sites in the region.
